# Electrochemical Reduction of La_2_O_3_, Nd_2_O_3_, and CeO_2_ in LiCl-Li_2_O Melt

**DOI:** 10.3390/ma15113963

**Published:** 2022-06-02

**Authors:** Alexey V. Shishkin, Vladimir Yu. Shishkin, Aleksandr A. Pankratov, Anna A. Burdina, Yuriy P. Zaikov

**Affiliations:** Institute of High Temperature Electrochemistry, Ural Branch, Russian Academy of Sciences, 620066 Ekaterinburg, Russia; v.shishkin@ihte.uran.ru (V.Y.S.); a.pankratov@ihte.uran.ru (A.A.P.); annaburdina89@gmail.com (A.A.B.); dir@ihte.uran.ru (Y.P.Z.)

**Keywords:** rare-earth metals, reduction, electrolysis, bromide method, reduction melting, metallization

## Abstract

The reduction of pellets composed of individual CeO_2_, Nd_2_O_3_ and a La_2_O_3_-Nd_2_O_3_-CeO_2_ mixture by lithium extracted on a cathode during lithium chloride electrolysis at 650 °C was studied. The methods of cyclic voltammetry, electron microscopy, including determination of the elemental composition of the studied objects, and X-ray diffraction analysis were applied for the present study. The reduction degree of rare-earth metal (REM) oxides was determined using both the bromine method and reduction melting of the samples in the graphite crucible. The formation of the metallic phase composed of the rare-earth elements (REEs) was not observed even at the cathode potentials, corresponding to the formation of the liquid alkali metal phase, and lithium extraction, which, in the quantitative ratio, exceeds greatly the values needed for the reduction reaction. CeO_2_ was found to reduce to Ce_2_O_3_.

## 1. Introduction

The operation of fast neutron reactors is inextricably related with the development of the closed nuclear fuel cycle based on the processing of highly active spent nuclear fuel (SNF) and returning uranium and plutonium fuel back to operation [1]. Pyrochemical methods are considered as a means to separate fission products [2,3]. Rare-earth metals (REMs) may be a distinguished group of fission products, as their chemical properties are extremely similar to the target products, i.e., uranium and plutonium. This fact determines technological operations and methods of their separation. Currently, extensive research related to the usage of molten salts as media for SNF processing is performed [4,5]. The reduction of oxide SNF by lithium is one of the research directions. Metallic lithium is extracted during the electrolysis of the molten lithium oxide-chloride mixture at 650 °C [6,7,8,9]. In our opinion, the behavior of a number of REM oxides in this process, i.e., their possible reduction to metal, together with uranium, for instance, still remains uncertain. Thus, based on the thermodynamic analysis of the interactions between metallic lithium and uranium oxide (reaction (1)) and, for example, metallic lithium and lanthanum and neodymium oxides (reactions (2) and (3)), we may conclude that lanthanum and neodymium oxides are not supposed to be reduced by lithium, as opposed to uranium oxide.

1/2UO_2(s)_ + 2Li_(l)_ → 1/2U_(s)_ + Li_2_O_(s)_; ΔG _650 °C_ = −13.38 kJ (1)

1/3La_2_O_3(s)_ + 2Li_(l)_ → 2/3La_(s)_ + Li_2_O_(s)_; ΔG _650 °C_ = +34.47 kJ (2)

1/3Nd_2_O_3(s)_ + 2Li_(l)_ → 2/3Nd_(s)_ + Li_2_O_(s)_; ΔG _650 °C_ = +39.70 kJ (3)

Uranium oxide was found to be completely reduced to metallic uranium [6,7,8,9]. However, a number of researchers have reported on the significant (about 60% or greater) reduction of REM oxides, including lanthanum and neodymium, to metals in SNF [10,11]. This contradiction may be explained by the erroneous interpretation of the “bromine method” used for the determination of the mass fraction of the REM oxides reduced by metallic lithium [12,13,14,15]. The analysis of the metallic phase in the simulated nuclear fuel requires a reasonable approach to select an appropriate method for the samples analysis, because the analyzed product is normally composed of a number of metal oxides, which imitate the SNF fission products.

This paper is devoted to the analysis of the reduction processes of individual CeO_2_ and Nd_2_O_3_, as well as the La_2_O_3_-Nd_2_O_3_-CeO_2_ mixture without the presence of any other metal oxides.

The following reaction takes place at the cathode during the electrolysis of the LiCl-Li_2_O melt:

Li^+^ + e → Li (4)

Extracted lithium interacts with REM oxide; for instance, with Nd_2_O_3_, according to reaction (3), lithium oxide dissolves in the chloride melt. A decrease in the lithium oxide concentration shifts the equilibrium of the system toward the formation of rare-earth metals. That is why pure lithium chloride was chosen as the initial melt for the first test run. A glassy carbon rod was used as an anode, where the following reaction takes place:

2Cl^−^ − 2e → Cl_2(g)_ (5)

From then on, a dense ceramic anode based on nickel oxide doped by lithium oxide was used in the oxide-chloride melt [15,16], and small amounts of lithium oxide (1.0–1.5 wt.%) were added to the melt. Therefore, the anode reaction acquired the following form:

2O^2−^ − 4e → O_2(g)_ (6)

## 2. Experiment

### 2.1. Reagents

Chemically pure anhydrous lithium chloride containing 99.3 wt.% of the basic component was used. To remove water traces, the salt was hermetically sealed, heated stepwise, and melted in an argon atmosphere. To remove the remaining oxygen admixtures, we used the method of zone melting in a nickel boat under the flow of argon purified from oxygen traces and water vapors [17]. Chemically pure lithium oxide containing more than 99.5 wt.% of the base component was analyzed to determine the concentrations of water, lithium hydroxide, and carbonate by infrared spectroscopy using a «FTIR» TENSOR 27 device (Bruker, Borken, Germany). The summarized concentration of admixtures did not exceed 0.05 wt.%.

The REM oxide pellets had a porosity of 40–50% and the following dimensions: height of 2–4 mm and diameter of 10.1–10.2 mm. The pellets were pressed from the REM oxide powder of the 5 ÷ 20 µm fraction under the pressure of 370–650 MPa. Zinc stearate was used as a binding agent. Then, the pressed pellets were annealed in air at a stepwise heating rate of 150–200 °C per hour up to 1200 °C and were exposed for 2 h at this temperature.

### 2.2. Electrolytic Cell

**Figure 1:**
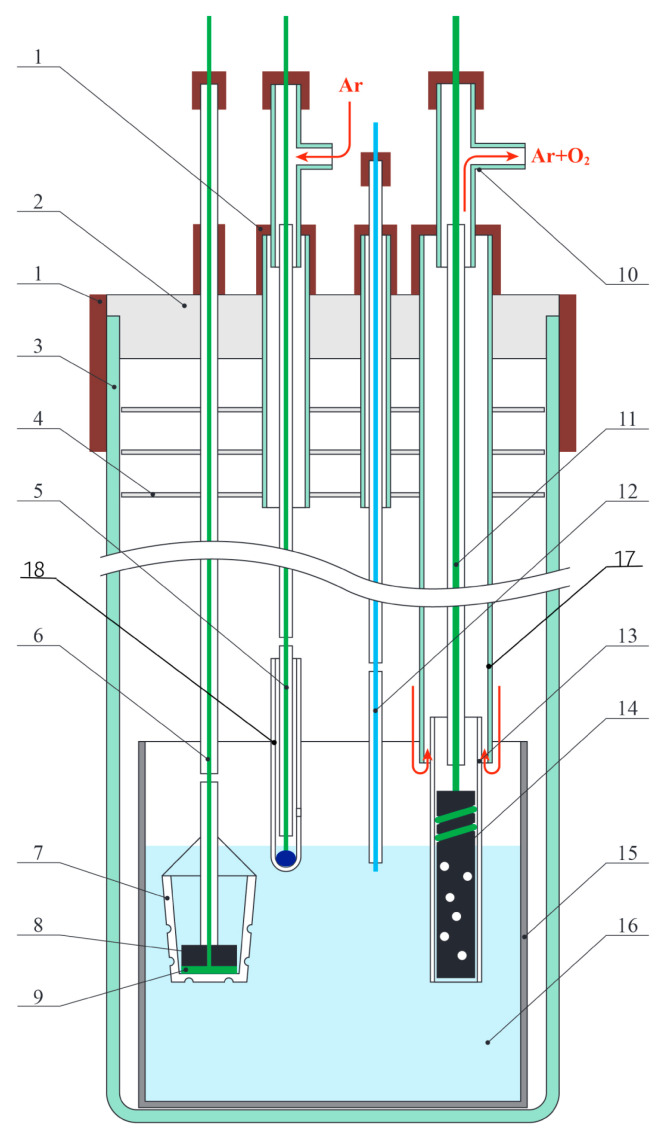
Schematic of the laboratory electrolytic cell: **1**—silicon rubber seals; **2**—fluoroplastic cover; **3**—electrolytic cell body; **4**—thermal screen; **5**—Bi-Li reference electrode; **6**—Mo current lead; **7**—MgO ceramic crucible; **8**—REM oxide pellet; **9**—Mo wire cathode; **10**—T-shaped glass pipe; **11**—Pt current lead to the anode; **12**—auxiliary Mo electrode; **13**—ceramic MgO tube, which covers the anode; **14**—NiO-Li_2_O (glassy carbon) anode; **15**—metallic (stainless steel or nickel) or MgO ceramic container for the electrolyte; **16**—LiCl-Li_2_O electrolyte; **17**—quartz glass tube; **18**—MgO ceramic tube.

Figure 1 illustrates the schematic of the electrolytic cell. The cell body was made of a quartz glass cylinder of 50 cm in height and 9 cm in diameter. A container made of MgO ceramics or stainless steel or nickel (**15**) was put on the bottom of the cylinder. A mixture of LiCl-Li_2_O (0.0–0.1 wt.%) amounting to 200–300 g was loaded into the container. Depending on the purpose of the experiment, either a ceramic or metal container was used. For example, in experiments on the Nd_2_O_3_ pellets reduction in the melt without initial lithium oxide content (0 mol.%), a ceramic MgO container and the glassy carbon anode were used. Chlorine gas was released on the glassy carbon anode.

The anode assembly was made of two tubes inserted into each other: the inside tube (**13**) was made of high-purity dense MgO ceramics and the outer tube (**17**) was made of quartz glass. The outer tube was installed into a fluoroplastic cover (**2**) of the electrolytic cell. A glassy carbon rod of 6 mm in diameter or a rod of 10 mm in diameter composed of gas-resistant NiO-Li_2_O ceramics served as an anode (**14**). The anode was located inside the MgO ceramic tube. The anode part was partially immersed into the electrolyte (**16**). A platinum wire of 1.00 mm in diameter served as a current lead (**11**) to the NiO-Li_2_O ceramic anode. The wire was fixed in a special cavity in the upper part of the anode. Different types of anodes were chosen for different experiments depending on the problem to solve.

A circle metallic nickel plate or intercoiled Mo wire served as a cathode (**9**). The cathode was placed on the bottom of a MgO ceramic crucible (**7**). A molybdenum wire current lead (**6**) to the cathode was used. Small openings in the side walls of the crucible facilitated the electrolyte diffusion through its walls. To perform the electrochemical measurements, mainly, to measure the cathode and anode potentials during the electrolysis or other manipulations, we used a Bi-Li (60 mol.%) reference electrode (**5**). Bismuth was placed in a MgO ceramic tube (**18**) with a small opening in the bottom part. We used a Mo wire current lead to the reference electrode. The alloy was prepared directly in the electrolytic cell before the electrochemical reduction of REM oxides. When the electrolytic cell was assembled and the electrolyte was melted, the reference electrode was immersed into the melt to provide contact with the electrolyte through an opening in the MgO tube (**18**) illustrated in Figure 1. The anode was immersed into the melt and the electrolysis was performed at 650 °C to extract lithium on liquid bismuth located in the MgO ceramic tube (**18**). When the reference electrode was formed, it was lifted so that the openings in the ceramic tube were above the melt surface [18].

To determine the value of the potential of the prepared alloy of the reference electrode relative to the lithium extraction potential, we immersed an auxiliary electrode made of Mo wire of 1.0–1.5 mm in diameter and 0.3–0.5 cm^2^ surface area into the melt. The voltammetry curves were recorded when the auxiliary Mo electrode functioned as a working electrode, the anode served as an auxiliary electrode, and the Bi-Li alloy served as a reference electrode. The reference electrode was calibrated relative to the lithium extraction potential. The value of the reference electrode potential during the long-term experiments on the electrochemical reduction of lanthanide oxide and after complete reduction of each cathode was analogously monitored. All parts of the electrolytic cell were hermetically assembled to the fluoroplastic cover of the electrolytic cell using vacuum-dense silicone rubber seals. All assembling operations, procedures with lithium chloride and oxide, were performed in a glove box in argon atmosphere at an oxygen content not exceeding 10 ppm and water vapors not exceeding 0.1 ppm.

The electrolysis was performed under the galvanostatic regime. The process parameters were set and controlled using a «Potentiostat/Galvanostat/ZRA/Referance 600» (Gamry Instruments, Inc., Warminster, PA, USA) and «PGSTAT AutoLab» (Ecohemie, Utrecht, The Netherlands). The anode potential was controlled by a universal digital voltmeter GDM-78351 (GW Instek, Taiwan, China). The electrolyte was sampled during the experiment; the concentration of lithium oxide was determined in situ by the acid–base titration method. The chemical analysis of the solutions was performed by an emission spectrometer with the inductively coupled plasma, «Optima 4300 DV» («Perkin Elmer», Waltham, MA, USA). The bromine method was used to determine the reduction degree of oxides [14]. Apart from that, the concentration of oxygen residues in the reduced product was determined after the removal of the electrolyte by the vacuum distillation. To analyze the oxygen concentration, LECO OH836 (LECO, St. Joseph, MI, USA) and METAVAK-AK (Eksan, Saratov, Russia) devices were used. The data obtained using both devices supplemented the measured results. These devices allowed obtaining more reliable data at different oxygen concentrations.

## 3. Results and Discussion

**Figure 2:**
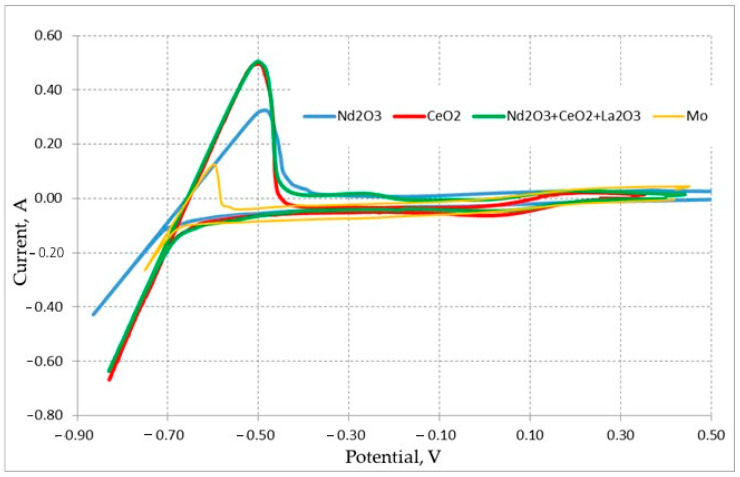
Cyclic voltammograms of REM pellets located on the Mo electrode recorded in the LiCl—Li_2_O (0–0.8 wt.%) melt at 650 °C. The scan rate was 0.01 V/s. The Bi-Li reference electrode was used (0.655 V relative Li^+^/Li).

First, we determined the potential of the reference electrode relative to the potential of lithium extraction on the Mo electrode. Then, we recorded the voltammograms of the cathode samples composed of individual Nd_2_O_3_ (described by reaction (3)), CeO_2_ oxides, and their La_2_O_3_-Nd_2_O_3_-CeO_2_ mixture, as well as the voltammograms of the auxiliary Mo electrode. The obtained results are illustrated in Figure 2. All curves demonstrated a clear peak in the anode region, which corresponded to the dissolution of lithium, extracted in the cathode region.

**Figure 3:**
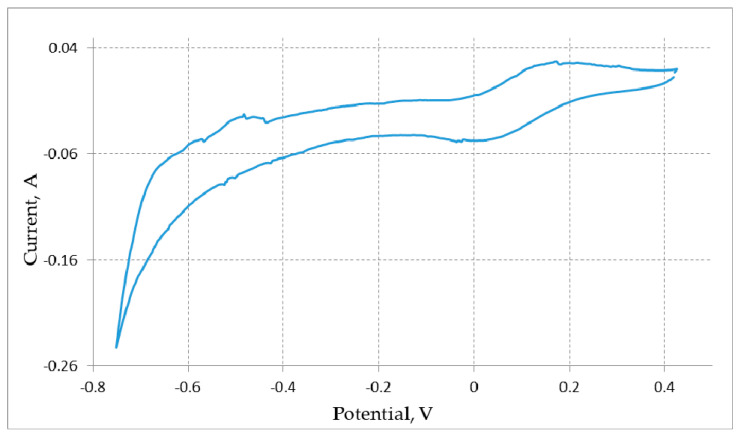
Cyclic voltammograms of the UO_2_ + (CeO_2_ + La_2_O_3_ + Nd_2_O_3_) 5 wt.% pellet located on the Mo electrode recorded in the LiCl—Li_2_O (1.0 wt.%) melt at 650 °C. The scan rate was 0.01 V/s; the Bi-Li reference electrode (0.700 V relative to Li^+^/Li) was used.

Figure 3 illustrates the analogous cyclic voltammogram recorded for the uranium dioxide pellet containing 5 wt.% of REM oxides. It is seen that there was no anode peak of the cathode lithium dissolution. This is associated with the fact that extracted lithium was consumed during the reaction of uranium oxide reduction (1) to uranium metal.

**Figure 4:**
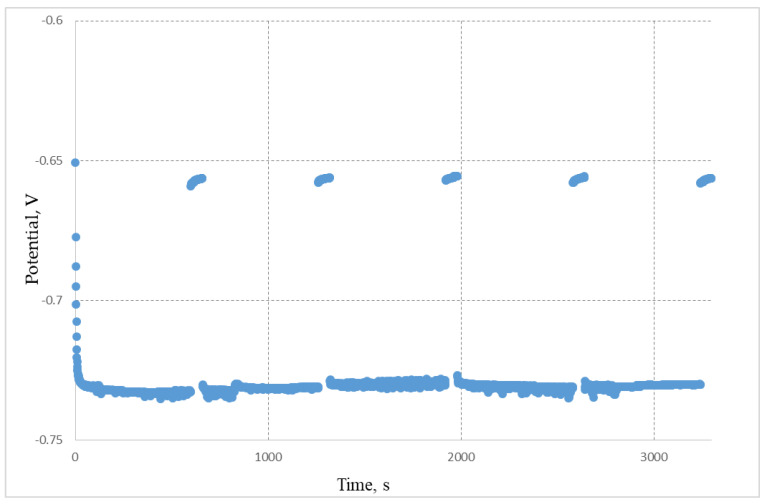
Change in the cathode potential during the electrolysis of the pellets composed of REM oxides in the LiCl—Li_2_O (0.5 wt.%) melt. The current of 0.3 A was passed through the melt during 600 s and then was interrupted for 60 s. The cycles were repeated. Upper curves denote the cathode potential relative to the Bi-Li reference electrode (0.658 V relative to Li^+^/Li) at the electrolysis current interruptions. Lower curves denote the cathode potential under the current load; 15% of the theoretical value of the electric current was passed through the melt.

Figure 4 demonstrates the change in the cathode potential during the electrolysis under the galvanostatic regime at the reduction of the pellet containing REM oxides. The cathode potential did not change in time after the current switch off and it was equal to the potential of the liquid lithium phase formation. This elucidates that the extracted lithium was not consumed for the REM oxides reduction.

Parameters and regimes of the experiments on the electrochemical reduction of REM oxides are presented in Table 1.

Table 1 illustrates that the experiments on the reduction of REM oxide pellets were performed at different concentrations of Li_2_O in the melt ranging from 0.0 to 0.8 wt.% and at different amounts of the electric current (Q) passed ranging from 136 to 201% of the theoretical value (Q_theor_ calculated according to reactions (2) and (3)).

**Figure 5:**
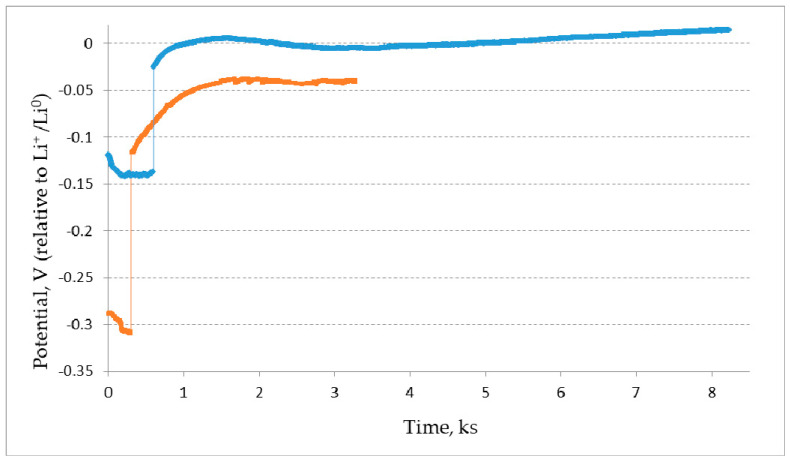
Change in the cathode potential relative to Li^+^/Li^0^ at the current interruption after 300 s (orange) and 600 s (blue) of the electrolysis at the current load of –0.4 A and –0.25 A, respectively. Q_exp_/Q_theor_ is equal to 17% and Q_exp_/Q_theor_ is equal to 136%. A Nd_2_O_3_ pellet was used.

The Nd_2_O_3_ pellets were reduced in the pure LiCl melt. Lithium oxide was not added to the initial melt. In this process, the MgO cathode container and glassy carbon anode were used. During the reduction process, gaseous chlorine evolved on the glassy carbon anode. The changes in the cathode potential are illustrated in Figure 5. It was well seen that the cathode potential remained unchanged during the electric current interruptions and it remained equal to the potential of lithium extraction.

**Figure 6:**
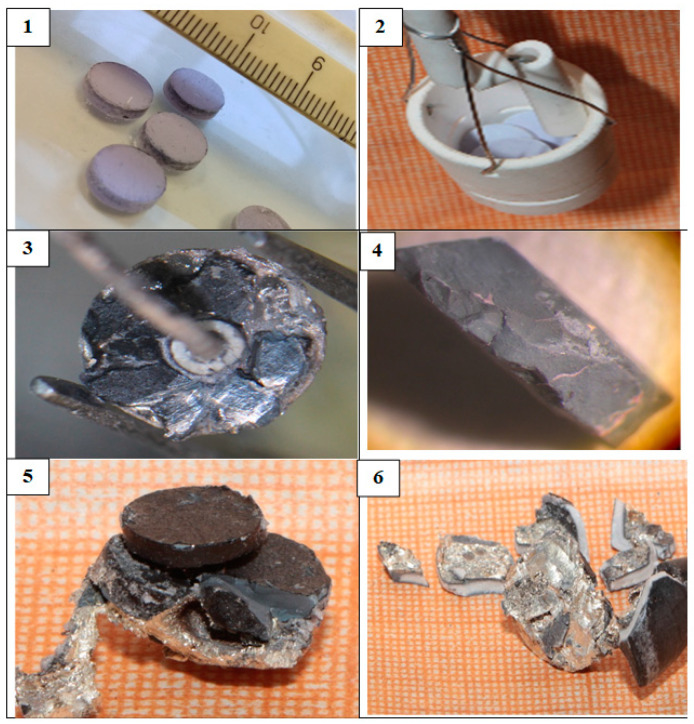
(**1**) Pressed and annealed Nd_2_O_3_ pellets; (**2**) Nd_2_O_3_ pellets loaded into the cathode crucible; (**3**) mechanically treated fragment of the Nd_2_O_3_ pellet on the nickel cathode after the electrolysis; (**4**) ethanol-rinsed and dried fragment of the Nd_2_O_3_ pellet; (**5**) top view of the Nd_2_O_3_ pellet after the reduction on the Mo current lead separated from the cathode crucible; (**6**) bottom view of the Nd_2_O_3_ pellet after the reduction on the Mo current lead separated from the cathode crucible.

Figure 6(1) illustrates the photograph of the Nd_2_O_3_ pellets before the electrolysis. The fragments of the pellets on the Ni cathode after the electrolysis are presented in Figure 6(2). The pellet was divided into fragments before the electrolysis to place it into the Ni cathode. A shiny metallic coating was observed at the pellet surface and current lead after the electrolysis; this coating was clearly seen after light mechanical treatment of the pellet (Figure 6(3)). The pellet fragment was rinsed in distilled water. The solution was first titrated by a weak solution of hydrochloric acid to determine the alkaline hardness and then the elemental chemical analysis was performed. During the analysis, it became clear that the metallic coating at the pellets surface was extremely thin and, during the dissolution of metallic coating, hydrogen bubbles evolved.

**Figure 7:**
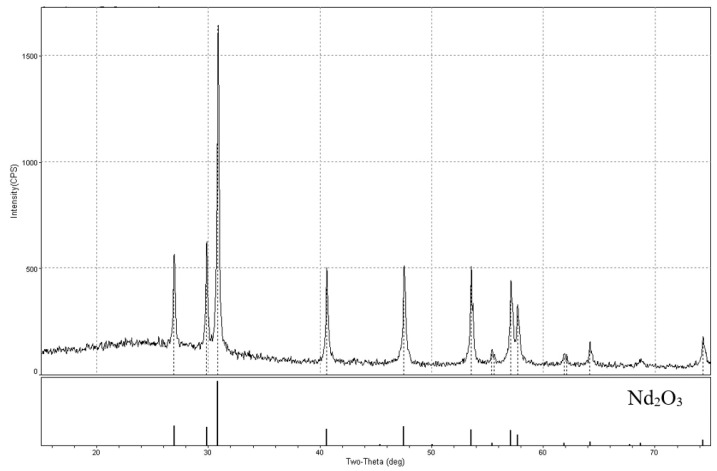
X-ray diffraction pattern of the ethanol-rinsed Nd_2_O_3_ pellet after the electrolysis.

Figure 6(4) illustrates the photograph of the ethanol-rinsed and dried fragment of the Nd_2_O_3_ pellet. Figure 7 presents the X-ray diffraction pattern of this fragment, which was identical to the XRD pattern of the Nd_2_O_3_ pellet recorded before the electrolysis. This fact testifies that the analyzed Nd_2_O_3_ pellet fragment did not undergo any changes.

We performed a chemical analysis of the metallic films that covered the pellets surfaces and the current lead as well as the surface of the ceramic insulator of the current lead to the Ni cathode and the surface of the MgO ceramic crucible under the Ni cathode. The films were dissolved in water. The combination of the elemental chemical analysis and X-ray diffraction data brought us to the conclusion that under the experimental conditions, the MgO ceramics was reduced instead of the Nd_2_O_3_ pellet and, hence, complex alloys containing mainly lithium, magnesium, and nickel formed.

**Figure 8:**
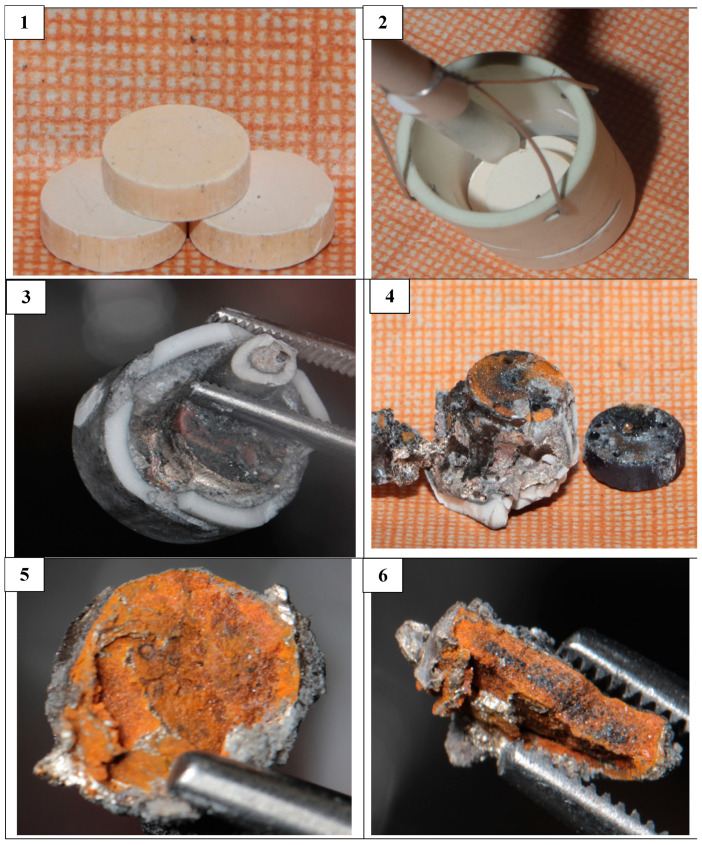
(**1**) Pressed and annealed CeO_2_ pellets; (**2**) CeO_2_ pellets in the cathode crucible; (**3**) CeO_2_ pellets in the Mo cathode after the electrolysis; the crucible walls were broken; (**4**) pellets separated from the ceramic crucible together with the Mo current lead; (**5**) lengthwise fault of the CeO_2_ pellets after the reduction; and (**6**) transverse fault of the CeO_2_ pellets after the reduction. The initial porosity of the pellets was 20%.

**Figure 9:**
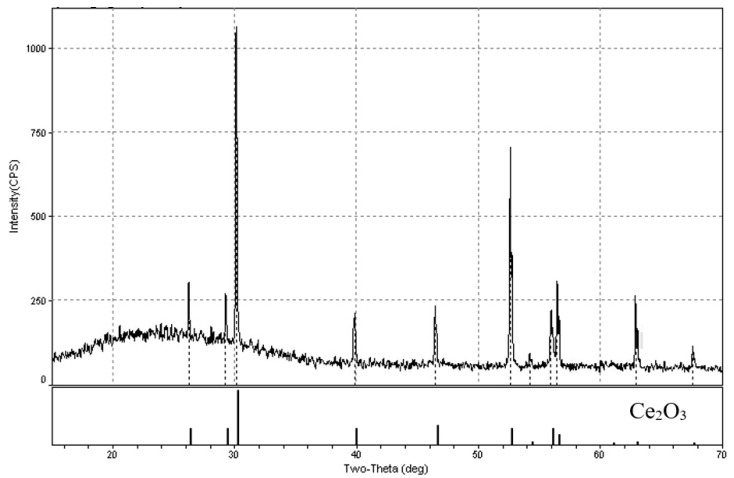
X-ray pattern of Ce_2_O_3_, obtained after the CeO_2_ pellet reduction.

Figure 8 presents the photographs of CeO_2_ pellets before and after the reduction. The samples after the reduction had mainly a yellow-orange or brown color, which are typical for Ce_2_O_3_ [19,20]. This fact is testified by the X-ray diffraction pattern presented in Figure 9.

**Figure 10:**
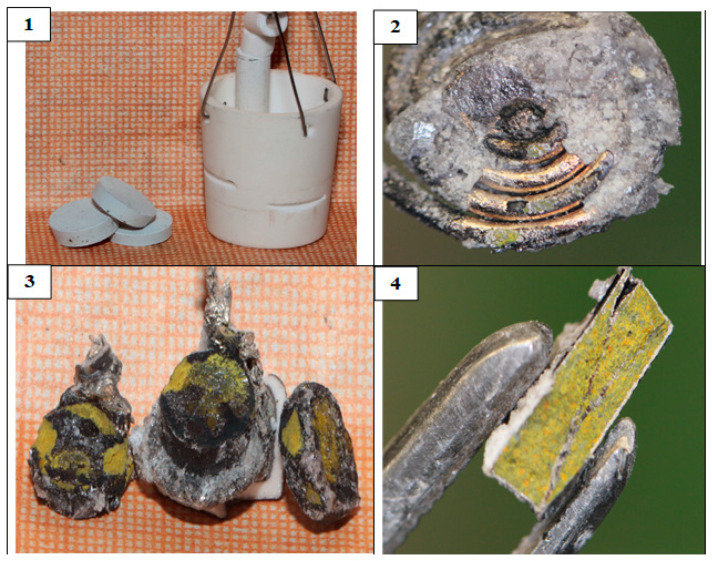
(**1**) Pressed and annealed La_2_O_3_-CeO_2_-Nd_2_O_3_ pellets (1:1:1 ratio) and MgO ceramic cathode crucible; (**2**) Mo cathode, where the pellets were put during the electrolysis; (**3**) REM oxide pellets after reduction and the Mo current lead, separated from the ceramic crucible; (**4**) transverse fault of the REM oxide pellet after the reduction.

Figure 10 demonstrates the reduced pellet composed of the La_2_O_3_-CeO_2_-Nd_2_O_3_ mixture in the 1:1:1 ratio. Figure 10(1) illustrates the pellet fragment before the experiment and Figure 10(4) on the right presents the transverse fault of the pellet. The presence of a yellow color elucidates the Ce_2_O_3_ oxide formation.

In the aforesaid experiments, the bottom part of the Mo current lead was intercoiled, as presented in Figure 10. It was seen that after the experiments both with CeO_2_ and with Nd_2_O_3_, the current lead was covered with metallic film composed of lithium and magnesium alloy.

**Figure 11:**
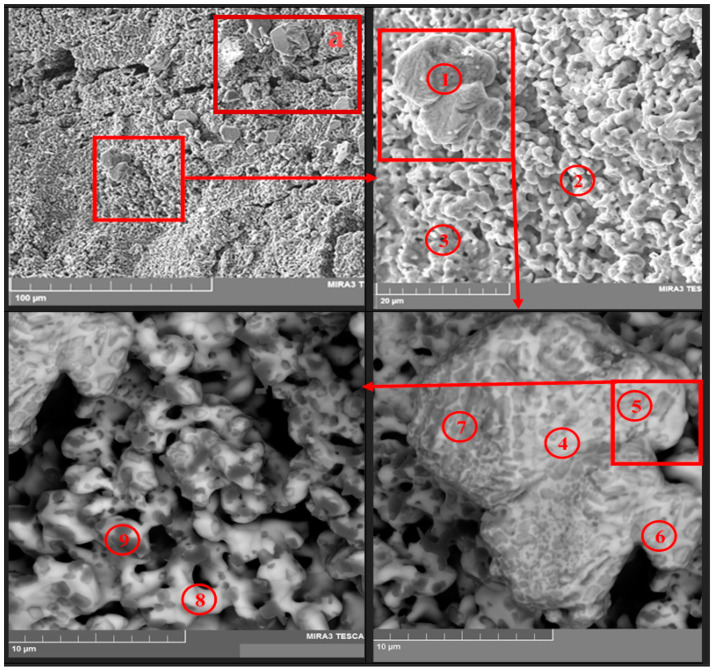
SEM images of the fragment of the La_2_O_3_-CeO_2_-Nd_2_O_3_ pellet after the electrolysis and distillation of the electrolyte products. The numbers illustrate the regions of the sample taken for the elemental analysis (see Table 2).

**Figure 12:**
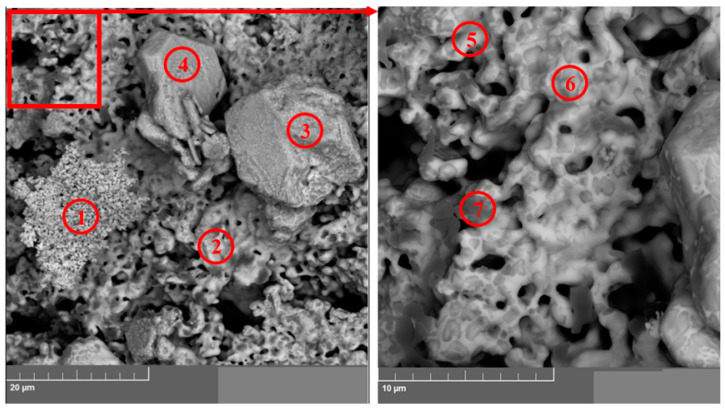
SEM images of the region (a) illustrated in Figure 11. The numbers illustrate the regions of the sample taken for the elemental analysis (see Table 3).

After the vacuum distillation of lithium chloride from the pellet surface, fragments of the samples from different experiments were analyzed using a TESCAN MIRA 3 LMU microscope (TESCAN, Brno, Czech Republic). The results of the analysis are provided in Figure 11 and Figure 12 and Table 2 and Table 3.

Figure 11 presents SEM images of the fragment of the La_2_O_3_-CeO_2_-Nd_2_O_3_ pellet after the electrolysis and subsequent distillation in the resolution of 100 µm, and Figure 12 illustrates the upper right part of the SEM image presented in Figure 11a at a resolution of 20 µm. The data on the elemental analysis of the pellet fragment is presented in Table 2 and Table 3. The regions of the pellets sampled for the elemental analysis are marked with numbers in Figure 11 and Figure 12.

The analysis of the results obtained using the TESCAN MIRA microscope (TESCAN, Czech Republic) and those reported in Table 2 and Table 3 illustrate that the studied samples were composed only of REM oxides; the concentration of oxygen in them was close to that in lanthanide sesquioxides. A significant amount of metallic magnesium was determined. Obviously, this was due to the partial reduction of MgO ceramic cathode containers.

The obtained results on CeO_2_ reduction are of great importance as they allow us to identify them with the PuO_2_ reduction. According to the thermodynamic analysis, CeO_2_ reduction is possible [21] and, therefore, the metallization process is supposed to proceed to metal extraction. This reaction is even more preferable than the metallization of UO_2_.

0.5CeO_2(s)_ + 2Li_(l)_ → 0.5Ce_(s)_ + Li2O_(s)_ ΔG _650 °C_ = −26.67 kJ (7)

1/3Ce_2_O_3(s)_ + 2Li_(l)_ →2/3Ce_(s)_ +Li_2_O_(s)_ ΔG _650 °C_ = 40.02 kJ (8)

2CeO_2(s)_ + 2Li_(l)_ → Ce_2_O_3(s)_ + Li_2_O_(s)_ ΔG _650 °C_ = −226.73 kJ (9)

However, it was experimentally shown that cerium reduced only up to the cerium sesquioxide, and further reduction to metal did not take place. Therefore, the oxides reduction proceeded subsequently via oxides with the smallest oxygen concentration, and the strongest oxide would become the limiting link of the chain. Thus, we may predict to a certain extent the possibility of PuO_2_ reduction. It is important to understand that the end product of the PuO_2_ reduction may be composed of metallic Pu and plutonium sesquioxide, in the case when the reduction process is incomplete. This is vital for determination of the PuO_2_ reduction by the bromine method.

It was found that the CeO_2_ reduction to its sesquioxide is highly probable, for instance, it is more thermodynamically likely than that of UO_2_ reduction to metal (reactions (1) and (9)). In the case of uranium, the anode peak of the extracted lithium dissolution was clearly absent on the voltammetry curves, which is explained by its consumption during UO_2_ reduction. The analogous picture was not observed for cerium. We suppose that this fact may be associated with the formation of a separate individual metallic uranium phase, where lithium was extracted; this process, obviously, also took place in the volume of the porous sample. Even at the reduction of highly dense UO_2_ samples, a porous layer of the metallic uranium phase, which did not prevent the following reduction, was formed at their surfaces. The processes related with cerium are more complex: cerium dioxide transforms into intermediate oxides (CeO_1.81_, CeO_1.71_, CeO_1.68_, and CeO_1.66_) [19], which form solid solutions with insignificant increases in volume. All these solid solutions are dielectrics or, at best, semiconductors. Thus, the rate of reaction is determined by the diffusion of the reduced forms of lithium into the pellet bulk and oxygen diffusion in the solid phase of cerium oxides, which results in the slower consumption of lithium as opposed to the process with uranium.

It is crucial to determine the reduction degree (β) of the REM oxide samples, i.e., to determine the relation of the formed metallic phase to the total amount of REM metal in the sample.

β = 100 × m_(Ln−Ln)_/(m_(Ln−Ln2O3) +_ m_(Ln−Ln0)_), (10)

where m_(Ln−Ln)_ is the weight of the lanthanide in the metallic phase and m_(Ln-Ln2O3)_ is the weight of lanthanide in the oxide phase.

A bromine method is recognized in many papers as promising for analyzing the metallic uranium-uranium dioxide system [12,13,14]. A metallic phase is known to dissolve well in a solution of bromine in ethyl acetate, whereas the oxide phase remains in the deposit, which is a prerequisite for the separation of metallic and oxide phases. We determined the reduction degrees of neodymium and cerium oxides after the electrolysis using the dissolution conditions of the reduced oxide samples provided in [14]. The obtained values are presented in Table 4.

It is seen that the studied samples may contain up to several wt.% of REM metallic phase. However, the X-ray diffraction patterns illustrated only cerium and neodymium oxide phases. In addition, this was characteristic both for the pellets containing the electrolyte and those rinsed in dehydrated alcohol or distilled in vacuum. In all cases, the X-ray diffraction patterns were recorded in hermetically sealed sample holders (Rigaku, Tokyo, Japan) with an inert atmosphere. The fragments of the pellets after the vacuum distillation of the captured electrolyte were studied using the electron microscope and their elemental composition was determined. It was found that cerium and neodymium appeared to be bound only with oxygen. The concentration ratio was close to the theoretical one for the corresponding oxides. Therefore, the data obtained using the bromine method contradicted with the data obtained using the other mentioned methods. That is why, using the bromine method, we determined the amount of metallic and oxide phases in the model objects, composed of the known amount of each phase (Table 5), and in the model samples, composed solely of neodymium oxide (Table 6). The experimental conditions were identical to those reported in [14].

The obtained results illustrate that the bromine method used to determine the degree of REM oxides reduction provided unstable and significantly inflated results. The dispersion degree influences the value of the reduction degree of oxides to metals when the bromine method is applied. That is why this method can be hardly used to analyze REM oxides, because of the difficulties associated with the results interpretation. The flawed data are mainly obtained at the analysis of oxide powders rather than highly dense pellets. However, after the lithium electrolytic reduction in the melt, simulated mixed oxide fuel and SNF pellets transform into the oxide powders. The thermodynamic analysis of the UO_2,_ Ce_2_O_3_, and Nd_2_O_3_ bromating reactions [21] elucidates to some extent the reason for the data misinterpretation:

1/2UO_2(s)_ + Br_2(l)_ → 1/2UBr_4(s)_ + 1/2O_2(g)_; ΔG _650 °C_ = +131 kJ, (11)

1/3Nd_2_O_3(s)_ + Br_2(l)_ → 2/3NdBr_3(s)_ + 1/2O_2(g)_; ΔG_650 °C_ = +11.9 kJ, (12)

1/3Ce_2_O_3(s)_ + Br_2(l)_ → 2/3CeBr_3(s)_ + 1/2O_2(g)_; ΔG_650 °C_ = +3.07 kJ, (13)

1/3La_2_O_3(s)_ + Br_2(l)_ → 2/3LaBr_3(s)_ + 1/2O_2(g)_; ΔG_650 °C_ = −14.7 kJ. (14)

In addition, we shall consider the fact that during the real experiment, the solutions of bromides in ethyl acetate are formed rather than their solid phases, as presented in equations. This promotes the shift in the equilibrium of reactions (11)–(14) to the right side.

Therefore, it becomes obvious that new methods for the reduced products analysis are required. That is why we determined the remaining oxygen content in the reduced sample, rather than the concentration of the obtained metal. We used the method of high-temperature melting of the sample in a graphite crucible. This method is well known. The sample is loaded into a graphite crucible and melted at high temperatures reaching 3000 °C in the flow of helium. During this process, the sample interacts with carbon, which results in the formation of gaseous carbon oxides CO_(gas)_ and CO_2(gas)_. The gaseous products are carried to the catalytic converter by the helium flow. Helium and carbon dioxide are the output gases from the catalytic converter. The concentration of carbon dioxide was quantitatively determined by the infrared spectrometer of LECO OH836 (LECO, USA) and METAVAK-AK (Eksan, Russia). First, the oxygen concentration in the individual CeO_2_ and Nd_2_O_3_ oxides samples was measured. The measurement results were in good agreement with the theoretical values. Then, we measured the oxygen concentration in mixed REM oxide samples. All samples preparation procedures were performed in a glove box in an inert argon atmosphere containing less than 10 ppm of oxygen and 0.1 ppm of water vapor. The measurement results are presented in Table 7, Table 8 and Table 9.

To calculate the concentration of oxygen in the samples, we assumed that after the reduction, CeO_2_ transforms into Ce_2_O_3_. The experimentally determined oxygen concentration in the reduced sample was in good agreement with the theoretical value, calculated following the aforesaid assumption.

Table 10 illustrates the calculated values of the oxygen concentration in the reduced samples, if we assume that the reduction degree is known. It is well seen that the oxygen concentration in the reduced sample is very sensitive to the degree of oxide reduction to metal.

Therefore, the provided calculation results and the experimentally obtained values of the oxygen concentration testify that La_2_O_3_, CeO_2_, and Nd_2_O_3_ are not reduced to metals during the electrolysis.

We should note that the pellets were impregnated with the electrolyte after the reduction. The weight of the mixed REM oxide pellets increased by 11%. Considering that the Li_2_O concentration in the melt did not exceed 0.7 wt.%, it may be easily calculated that oxygen added to the sample via alkali metal oxide, i.e., Li_2_O present in the electrolyte, changed the total oxygen concentration by less than 0.1%. This correction was furthermore less for the samples subjected to the vacuum distillation.

## 4. Conclusions

The electrochemical reduction of CeO_2_ and Nd_2_O_3_ as well as the mixture of La_2_O_3_-CeO_2_-Nd_2_O_3_ in lithium chloride melt with minimal LiO_2_ concentration was studied. It was experimentally found that the REM oxide pellets did not reduce to metals even at the Li_2_O concentration of zero. Lithium- and magnesium-containing alloys were formed as a result of the MgO ceramics metallization. Cerium dioxide was found to transform into its sesquioxide. The determination of the degree of metallization of the initial reagents during the reduction process is a key research parameter. However, this aspect requires further research and development. That is why, together with the known detection methods for determination of the metallic and oxide phases’ ratio in the uranium system, the method aimed at the determination of the remaining oxygen concentration in the end product was considered. The provided conclusions were verified by X-ray diffraction analysis, micro-X-ray analysis with scanning electron spectroscopy, elemental analysis, and high-temperature reduction melting of the sample in the graphite crucible.

## Figures and Tables

**Table 1:** Parameters and regimes of the REM oxides electrolysis.

Ln_2_O_3_	Pellet Weight, g	wt.% Li_2_O		E_Li^+^/Li^0^_, mV	I, A	Q_exp_/Q_theor_ %	Anode Material
		Before	After				
Nd_2_O_3_	1.26	0.00	0.03	−5 ÷ −60	0.10–0.35	161	glassy carbon
Nd_2_O_3_	1.27	0.03	0.07	−8 ÷ −110	0.20–0.40	136	glassy carbon
Nd_2_O_3_	2.19	0.86	0.71	+7 ÷ −6	0.20–0.40	191	Ceramics
CeO_2_	0.98	0.83	0.74	+85 ÷ 20	0.25–0.30	193	Ceramics
CeO_2_	2.62	0.74	0.44	+5 ÷ −2	0.25–0.40	162	Ceramics
Oxides mixture	0.69	0.77	0.55	+1 ÷ −2	0.10–0.30	166	Ceramics
Oxides mixture	1.11	0.55	0.49	+3 ÷ −1	0.15–0.30	184	Ceramics
Oxides mixture	2.53	0.52	0.28	+1 ÷ −1	0.20–0.40	201	Ceramics

**Table 2:** Data on the elemental analysis of the REM oxides pellet fragment sampled from points illustrated in Figure 11, atomic %.

Spectrum	O	Mg	Al	Cl	La	Ce	Nd
1	60.21	15.91	0.00	0.00	5.30	13.24	5.35
2	55.32	9.83	0.30	0.00	23.67	5.12	5.76
3	59.23	13.58	0.00	0.00	11.70	8.70	6.78
4	61.75	7.31	0.00	0.00	19.85	3.40	2.90
5	55.82	11.17	0.00	0.00	20.79	4.95	3.72
6	51.69	35.68	0.34	0.00	7.20	1.71	1.36
7	50.42	42.43	0.40	0.00	4.10	0.62	0.54
8	58.37	11.17	0.00	0.00	20.79	4.95	4.72
9	51.69	35.68	0.34	0.00	7.20	2.72	2.36

**Table 3:** Data on the elemental analysis of the REM oxides pellet sampled from different points illustrated in Figure 12, atomic %.

Spectrum	O	Mg	Cl	La	Ce	Nd
1	59.76	3.45	0.00	8.41	24.83	3.55
2	58.39	23.05	0.00	7.07	7.85	3.64
3	57.92	18.38	0.00	5.49	15.48	2.41
4	54.30	28.80	0.00	3.83	10.93	1.85
5	53.35	5.19	0.00	16.56	15.45	8.82
6	49.55	26.16	0.00	9.05	9.34	5.52
7	48.05	40.10	0.00	4.77	4.30	2.45

**Table 4:** Determination of the reduction degree of REM oxides to metals using the bromine method.

Sample	Q_exp_/Q_theor_ %	β, %		
Nd_2_O_3_	191	2.4		
CeO_2_	162	2.6		
Mixed	201	1.3 *	2.3 *	1.3 *

* β for La_2_O_3_, CeO_2_, Nd_2_O_3_, respectively.

**Table 5:** Concentrations of oxide and metallic REM phases determined using the bromine method in model samples.

Substance, g	1	2	3	4	5
La	0.048	0.039			
Nd	0.042	0.042	0.042	0.046	0.042
Ce		0.038			
La_2_O_3_	0.034	0.027			
Nd_2_O_3_	0.034	0.027	0.053	0.042	0.042
CeO_2_	0.034	0.031			
Actual degree of reduction, %	47.36	58.30	44.21	50.00	50.00
Analytical degree of reduction, %	94.89	98.30	95.12	98.30	94.81

**Table 6:** Concentration of oxide and metallic phases in model Nd_2_O_3_ samples determined by the bromide method.

№	Nd_2_O_3_ Sample, g	Actual Reduction %	Analytical Degree of Nd_2_O_3_ Reduction, %
1	0.155	0	4.95
2	0.153	0	10.86
3	0.155	0	9.28
4	0.160	0	57.81
5	0.167	0	44.01
6	0.52	0	38.80
7	0.51	0	67.20
8	0.52	0	44.99
9	0.51	0	54.06

**Table 7:** Calculated and experimental values of the oxygen concentration in mixed REM (La_2_O_3_-CeO_2_,-Nd_2_O_3_; 1:1:1) pellets before and after the reduction.

No.	Oxygen Concentration, wt.%				Q_exp_/Q_theor_ %
	Before Reduction		After Reduction		
	Calculated	Measured Using “LECO”	Calculated	Measured Using “LECO”	
1	15.70	15.5 ± 0.11	14.54	14.5 ± 0.14	166
2	15.70	15.7 ± 0.05	14.54	14.39 ± 0.05	184
3	15.70	15.3 ± 0.08	14.54	14.4 ± 0.12	201

**Table 8:** Calculated and experimental values of the oxygen concentration in the Nd_2_O_3_ pellets before and after the reduction.

No.	Oxygen Concentration, wt.%						Qexp/Qtheor %
	Before Reduction			After Reduction			
	Calculated	Measured Using “LECO”	Measured Using “Metavak”	Calculated	Measured Using “LECO”	Measured Using “Metavak”	
1	14.26	14.2 ± 0.03		14.26	14.2 ± 0.03		161
2	14.26	14.3 ± 0.12	14.41 ± 0.16	14.26	14.2 ± 0.03	14.55 ± 0.17	136
3	14.26	14.21 ± 0.10	14.37 ± 0.39	14.26	14.2 ± 0.03	14.35 ± 0.29	191

**Table 9:** Calculated and experimental values of the oxygen concentration in CeO_2_ pellets before and after the reduction.

No.	Oxygen Concentration, wt.%						Q_exp_/Q_theor_ %
	Before Reduction			After Reduction			
	Calculated	Measured Using “LECO”	Measured Using “Metavak”	Calculated	Measured Using “LECO”	Measured Using “Metavak”	
1	18.59	18.7 ± 0.13	18.91 ± 0.55	14.62	14.2 ± 0.03	14.69 ± 0.46	193
2	18.59	19.1 ± 0.12	18.86 ± 0.93	14.62	14.6 ± 0.28	14.72 ± 0.31	162

**Table 10:** Calculated values of oxygen concentration in reduced mixed REM oxide pellets considering that the reduction degree (β) is known.

Oxygen Concentration, wt.%	14.54	13.91	13.28	11.98	9.26
β, %	0	5	10	20	40

## Data Availability

Not applicable.
